# The various shades of ER‐phagy

**DOI:** 10.1111/febs.15031

**Published:** 2019-08-13

**Authors:** Alexandra Stolz, Paolo Grumati

**Affiliations:** ^1^ Structural Genomics Consortium, BMLS Goethe University Frankfurt Germany; ^2^ Institute of Biochemistry 2 Goethe University School of Medicine Frankfurt Germany; ^3^ Telethon Institute of Genetics and Medicine (TIGEM) Pozzuoli Italy

**Keywords:** autophagy, endoplasmic reticulum, ER‐phagy, lysosome

## Abstract

Endoplasmic reticulum (ER) is a large and dynamic cellular organelle. ER morphology consists of sheets, tubules, matrixes, and contact sites shared with other membranous organelles. The capacity of the ER to fulfill its numerous biological functions depends on its continuous remodeling and the quality control of its proteome. Selective turnover of the ER by autophagy, termed ER‐phagy, plays an important role in maintaining ER homeostasis. ER network integrity and turnover rely on specific ER‐phagy receptors, which influence and coordinate alterations in ER morphology and the degradation of ER contents and membranes via the lysosome, by interacting with the LC3/GABARAP family. In this commentary, we discuss general principles and identify the major players in this recently characterized form of selective autophagy, while simultaneously highlighting open questions in the field.

## Graphical abstract

The endoplasmic reticulum (ER) is a large and dynamic cellular organelle. Its capacity to fulfill the numerous biological functions depends on its continuous remodeling and the quality control of its proteome. Selective turnover of the ER (ER‐phagy) maintains ER homeostasis. ER network integrity and turnover rely on specific ER‐phagy receptors, which influence and coordinate alterations in ER morphology and the degradation of ER contents and membranes via the lysosome.

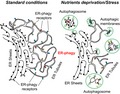

**Abbreviations**
ER: endoplasmic reticulum
ERAD: endoplasmic reticulum‐associated degradation
GABARAP: gamma aminobutyric acid receptor‐associated protein
IM: intramembrane
LC3: MAP1LC3 microtubule‐associated proteins 1A/1B light chain 3
LIR: LC3‐interacting region
mATG8: mammalian ATG8
TM: transmembrane
UIM: ubiquitin interacting motifs
UPR: unfolded protein response

## Introduction

The endoplasmic reticulum (ER) forms a continuous membrane network composed of sheets, tubules and matrices, which originate at the nuclear membrane and spread throughout the cytosol 1, 2, 3. Novel super‐resolution microscopy assays revealed that the ER, classically thought to be composed of sheets and tubules, actually consists of tubules of different densities. The apparent flat sheets are the result of very dense tubular matrices, which were described as sheets because conventional optical microscopy could not reach a high enough resolution to distinguish the tight clustering of tubular junctions 2. The various ER subdomains mediate a large number of vital cellular functions including Ca^2+^ homeostasis, protein synthesis, glycosylation, secretion, transport as well as lipid synthesis 4, 5. The ER is also a pivotal transportation hub for large number of intracellular and extracellular proteins: proteins destined for the plasma membrane, Golgi, extracellular matrix and many others travel to or through the ER to their destination. The abundance of individual ER structures within a cell correlates with cell type and tissue‐specific functions. Selective turnover of the ER by autophagy is termed ER‐phagy and plays a vital role in ER health and homeostasis. The impact of ER‐phagy on cellular homeostasis, its relationship to ER stress and unfolded protein response (UPR), its crosstalk with the other ER degradative mechanisms [ER‐associated protein degradation (ERAD) and ER to lysosome‐associated degradation], as well as its implications in human pathologies have been reviewed in depth in recent manuscripts 6, 7, 8, 9, 10, 11, 12, 13. In the mammalian systems, the term ER‐phagy is associated with the selective elimination of discrete fragments of the ER, enwrapped inside the autophagosomes and degraded via the lysosomal machinery. ER‐phagy has also different key functions depending on the cell type (e.g., neurons vs. pancreatic secretory cells) and cellular state (homeostasis vs stress). As such, ER‐phagy impacts on the quality, quantity, and secretion of ER proteins on one hand, and on the other hand molds the ER into a functional organelle (shaping ER subdomains in response to requirement and stresses). Despite the great interest that ER‐phagy is now arousing in the scientific community, it has been almost 40 years, since presence of ER membranes was first observed inside autophagosome. This type of selective autophagy remained in the shadow for decades and its molecular mechanisms just recently began to be unraveled.

## The history of ER‐phagy

**Figure 1:**
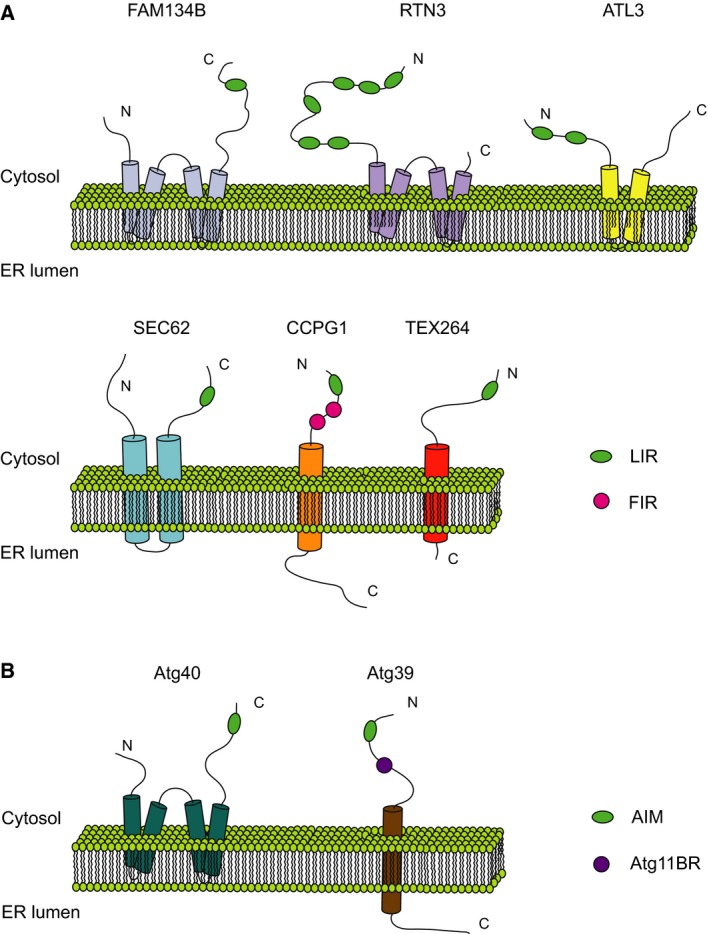
Schematic representation of ER‐phagy receptors in mammals and yeast. Abbreviations are (A) LIR (mammalian); FIR, FIP200‐interacting region (mammalian); (B) AIM, Atg8‐interacting region (yeast); Atg11BR, Atg11‐binding region (yeast). C, C‐terminal domain; N, N‐terminal domain.

It is now well accepted that macroautophagy (often synonymously termed autophagy) is not just a bulk degradative process, but is rather a selective mechanism able to isolate and eliminate specific cargo 14. In the case of ER‐phagy, autophagy cargo is defined as a discrete fragment of ER content (e.g., ER‐derived membranes with protein content). However, this view was not prevalent in the past decades. ER membranes were first documented inside a vesicular structure by Locke *et al*., in 1965 15. At that time, selective autophagy was still unknown, and the word ‘autophagy’ had just been introduced in 1963 by Christian de Duve. ER fragments inside autophagosomes were subsequently observed in rat hepatocytes after treatment with phenobarbital 16 and in guinea pig pancreatic cells 17. Over decades, alterations and dysfunctions in the ER were observed in cellular and animal models where the autophagy machinery was impaired 18, 19, 20. These phenotypes have mostly been attributed to a general impairment in macroautophagy. Moreover, in yeast, ER membranes were detected inside autophagosomes under starvation conditions. Even though this event was mainly attributed to a nonselective autophagic process, this marks the first time a selective engulfment of the ER was described 21. Immediately following this observation, microautophagy of ER whorls was found to contribute to the rescue of ER morphology following UPR‐induced ER membrane expansion in yeast. It is in this context that the term ER‐phagy arose for the first time 22, 23, 24, even though the underpinnings of this process were still unknown. Originally, interest in the ER, from the perspective of the autophagy field, could be mainly attributed to its role as a reservoir for autophagic membranes rather than itself being considered an autophagy target. Only in recent years, after the characterization of the first ER‐phagy receptors (see next paragraph), FAM134B in mammals and ATG39 and ATG40 in yeast 25, 26, the molecular basis of ER‐phagy was revealed and its full biological importance began to be recognized 10. Several mammalian ER‐phagy receptors have been identified so far: SEC62, RTN3 (its long isoform RTN3L), CCPG1, ATL3, and TEX264 27, 28, 29, 30, 31, 32 (Fig. 1A,B). The sheer number of ER‐phagy receptors, which will certainly only continue to rise, reflects the complexity of this selective form of autophagy; while, at the same time, unveiling the many shades and flavors of ER‐phagy, in different cell and tissue types as well as under a multitude of conditions.

## ER‐phagy receptors: different players, different substrates spectra, same purpose

**Figure 2:**
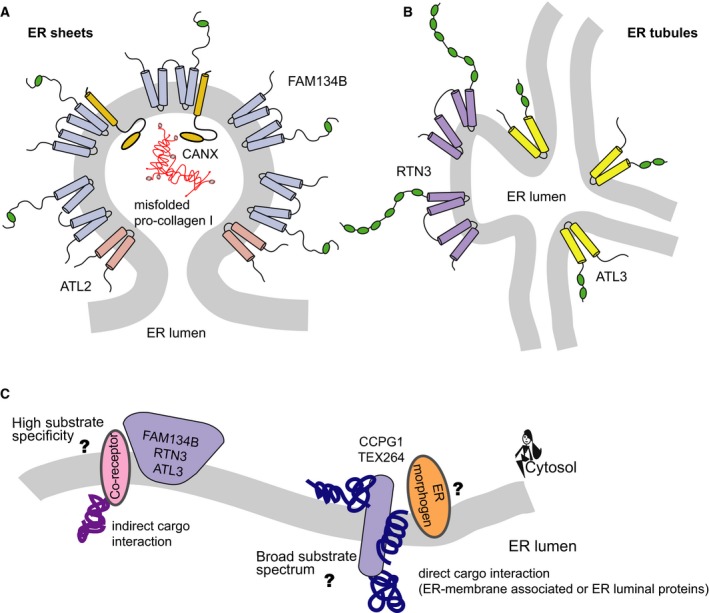
ER‐phagy receptors clustering, interaction and potential cargo recognition. (A) Schematic representation of FAM134B clustering and interaction with ATL2 for ER membrane vesiculation and CALNEXIN for misfolded procollagen recognition. (B) Schematic representation of RTN3 clustering and putative interaction with ATL3 for ER tubule degradation. (C) Hypothesis of how different ER chaperones could regulate specific cargo selection and elimination from the ER lumen. FAM134B, RTN3, and ATL3 may utilize co‐receptors for protein substrate binding and induce local ER fragmentation by clustering, while CCPG1 and TEX264 could directly interact with protein substrates and at the same time use other ER morphogenic proteins to separate ER fragments for lysosomal degradation.

Autophagy receptors, including the specific ER‐phagy ones, are defined as linkers between autophagic cargo and the MAP1LC3 microtubule‐associated proteins 1A/1B light chain 3 (LC3)/gamma aminobutyric acid receptor‐associated protein (GABARAP) family on autophagic membranes. By this, they facilitate cargo engulfment and its subsequent degradation via lysosome 33. As such, all ER‐phagy receptors harbor a functional LC3‐interacting region (LIR) and mediate ER degradation via lysosomes. These two common features aside, they have distinct structural domains, localize to different ER subdomains, act under different conditions (basal vs stresses), and focus on specific subsets of ER substrates (i.e. ER‐phagy receptors interact directly or indirectly, with protein aggregates inside the ER lumen to facilitate their degradation). This is because many of the identified receptors have a precise and well‐established (ER‐phagy independent) biological function at the ER, such as shaping ER membranes (i.e. FAM134B, RTN3L, ATL3). Taking a closer look at their structural characteristics, bona fide receptors are divided into intramembrane (IM) and transmembrane (TM) proteins. IM receptors are anchored to the ER membranes via a specific domain, (i.e. reticulum homology domain) inserted in the ER lipid bilayers. These receptors only face the cytosol, and do not have any structural domains in the intraluminal space of the ER. On the other hand, TM receptors span the entirety of the ER membrane with a portion of the protein facing the intraluminal space. IM proteins include: ATL3, FAM134B, RTN3L; and TM proteins include: CCPG1, SEC62, TEX264, respectively 3, 25, 27, 29, 30, 34, 35. Members of the IM group have well known functions in shaping ER subdomains, modulating tubule/sheet ratios as well as tubule branching 3, 35. In the case of FAM134B, biophysical and mathematical modeling indicates that this receptor can induce budding of membranes. As a consequence, the edges of ER sheets are enriched with FAM134B protein clusters and are hot spots for ER‐phagy 36. All three IM autophagy receptors are devoid of an ER luminal domain; therefore, one may argue that this limits their spectrum of protein substrates to ER trans‐ or intramembrane proteins. However, it is important to consider that ER membrane proteins with a luminal domain could function as autophagy adaptors. By simultaneously binding to protein aggregates, inside the ER lumen, and ER‐phagy receptors, such adaptors could be utilized as co‐receptors. These proteins can serve as a bridge allowing the inclusion of ER luminal protein aggregates in the substrate’s spectrum of IM ER‐phagy receptors, which themselves cannot directly interact with proteins inside the ER lumen. Indeed, such an adaptor has been identified in the case of procollagen, where the ER‐resident chaperone calnexin acts as a co‐receptor for FAM134B‐mediated ER‐phagy to prevent the accumulation of misfolded procollagen 37 (Fig. 2A).

Thus far, no intrinsic function (such as shaping of ER membranes) has been reported for any of the TM receptors. However, the TM receptors: CCPG1, SEC62 and TEX264, harbor cytosolic, ER‐membrane and ER‐intraluminal domains (a very small one for TEX264). Therefore, they are in principle capable of directly interacting with various ER‐localized proteins: ER luminal, membrane and even ER‐associated cytosolic proteins. This could potentially further divide ER‐phagy receptors into those facilitating bulk ER‐phagy, with a potential broad spectrum of ER elimination (entire portions of ER and a wide range of intraluminal ER protein aggregates), and those with higher selectivity for specific type ER domains or protein aggregates (Fig. 2C). Current knowledge partly supports this hypothesis. CCPG1, SEC62, and TEX264 all act under stress/stress‐resolving conditions such as protein aggregation, ER inflation, and nutrient deprivation 27, 29, 30, 32. Clearance of an overload of misfolded proteins, large amounts of membranes or ER contents could indeed be called bulk ER‐phagy and presumably depends on a receptor with a broad substrate spectrum. In contrast, dependency on co‐receptors, like in the case of FAM134B and misfolded procollagen 37, might speak to a more targeted and specific ER‐phagy receptor with a narrow substrate spectrum. Of note, RTN3 specifically regulates the elimination of prohormone aggregates, in particular *Akita* proinsulin aggregates, from the ER lumen 38. The molecular mechanisms are not completely clear; nevertheless, the involvement of a co‐receptor is a concrete possibility in this case too.

In yeast, only two ER‐phagy receptors have been identified so far: Atg39 and Atg40 26. Atg39 is present in the perinuclear region and Atg40 is responsible for cortical ER degradation. Any significant homology appears between these two receptors. While Atg40 has some similarities to FAM134B, Atg39 is a single‐pass TM protein similar in structure to CCPG1 and TEX264. Much like CCPG1, Atg39 can bind autophagy‐related proteins other than mammalian ATG8 (mATG8s: LC3s and GABARAPs). While Atg39 binds Atg11, CCPG1 interacts specifically with FIP200 26, 30.

Despite the differences between the various ER‐phagy receptors, there is one common feature: they contain an intrinsic disordered region, which generally has multiple functions including protein–protein interactions and membranes curving 39. So far, the intrinsic disordered region seems to be a common feature to almost all ER‐phagy receptors (this remains to be confirmed for ATL3) and this characteristic may be responsible for the ability of these proteins to attract autophagic membranes 27. As more ER‐phagy receptors are uncovered, likely with different ER sublocalizations, modes of action and substrates specificity, this hypothesis will be put to the test.

Of note, we still do not know which biological parameters define how much of the membrane is degraded, how this process is assisted and if there is a fixed protein/membrane ratio during the degradation of ER membrane and luminal proteins. It is also important to remember that, like TEX264, FAM134B and RTN3L have been reported to be active under starvation conditions and, for FAM134B and TEX264, the ER‐phagic activity was measured using the SS‐RFP‐GFP‐KDEL reporter 27. However, in contrast to TEX264, no quantitative mass spectrometry measurements have been performed for the other proteins 29. Therefore, we currently lack an unbiased, direct quantitative comparison of the contribution of the various ER‐phagy receptors to ER‐phagy and their substrate overlap/specificity under defined conditions.

## How is ER‐phagy activated and what kind of regulation levels do exist?

Identifying the signaling events which regulate ER‐phagy remains a major hurdle in the field due to the complexity of the ER in terms of its proteome, morphology and function in different cells and tissues. Thus far, efforts to identify a precise stressor or stimulus that ubiquitously induces ER‐phagy have been unsuccessful. Similarly, there are no common molecular pathways regulating ER‐phagy or at least they are still unexplored. Indeed, direct regulation of gene transcription by the UPR has, so far, only been observed for CCPG1 30.

So, how are ER‐phagy receptors ‘activated’? These receptors have no clear enzymatic activity; however, the binding of intraluminal ER protein aggregates or autophagic membranes to these receptors as well as their ability to cluster (as further explained below) could serve as surrogate measures for their activity. As mentioned above, in the case of CCPG1, regulation may occur on the level of transcription and/or translation 30. However, for most autophagy receptors expression does not seem to be the major regulatory step and therefore regulation must occur further downstream. Moreover, constant binding to LC3 and continuous turnover via autophagy, under basal conditions, has been shown for some autophagy receptors, such as cytosolic p62. It is unclear if this is accompanied by low‐level turnover of p62 substrates as well. It has also been shown that the binding affinity of p62 can be modulated by phosphorylation, so p62 activity depends on post‐transcriptional modifications (PTMs) 33. Considering that ER‐phagy can be triggered by starvation, some PTMs of receptors could change (i.e. phosphorylation state) and likely affect their activity.

Another feature of the ‘active’ state of an ER‐phagy receptors seems to be clustering. Clustering likely helps to maximize recruitment/tethering of adjacent autophagic membranes via mATG8s. Moreover, increasing the local density of ER‐phagy receptors, which are also ER morphogens (i.e. FAM134B and RTN3L) promotes ER membrane shaping to allow budding and fragmentation 31, 36. ER‐phagy receptors appear to work independently from one another and primarily form homodimers/multimers. The only exception, thus far, was reported for RTN3L and ATL3, which were found to interact with one another after protein overexpression in HEK293T cells 28. Although the atlastins family member, ATL2, has not been officially defined as an ER‐phagy receptor, ATL2 interacts with FAM134B. This interaction works to separate the ER buds marked by FAM134B oligomers from the rest of the ER body 40. The interaction between RTN3L and ATL3 could have a similar function and at the same time help to tether RTN3L to tubular junctions (Fig. 2A,B). In yeast, Lnp1, a protein found in three‐way junctions, is required to relocalize Atg40 from the cortex to the cell interior in order to promote the interaction between Atg40 and the autophagic machinery 41.

In summary, the field of ER‐phagy is still largely in its infancy and the underpinnings of this vital pathway are yet to be fully defined. The precise modifications, necessary interactions, cascades of events preceding the activation of the ER‐phagy receptors are all open questions, which remain to be answered. In principle, the following possibilities exist and, most probably, a combination of those takes place:

1. Activation via PTMs: PTMs can reveal or mask binding interfaces, which are necessary for the oligomerization, and modulation of receptor binding affinity to its substrates and to autophagic membranes.
2. Co‐receptors, auxiliary and scaffold proteins (i.e. ER chaperones or ER resident proteins): clustering as well as substrate binding could be facilitated via chaperones, this is likely the case with Calnexin in misfolded procollagen elimination 37. Multiple chaperones could serve the same receptor or recognize the same intraluminal ER protein aggregates. It is also conceivable that individual chaperones could bind to several ER‐phagy receptors. Both possible scenarios will increase the efficiency of ER cargo elimination. The regulation of such adapters may occur at several levels: gene expression, PTMs and protein–protein interaction. This is also relevant considering that not all of the receptors are equally expressed among different cell types and tissues. Of note, scaffold proteins, like Lnp1, can also anchor receptors to certain ER subdomains 42.
3. Local changes in the ER lipid bilayer (composition or modification of lipids) could allow and/or cause local enrichment of receptors. Changes in the lipid bilayer are also able to cause changes in protein structure 43, 44, which may influence oligomerization and binding interfaces for substrates/autophagic membranes. In addition, with the same principle, substrates could be relocalized to ER membrane crafts containing ER‐phagy receptors.

## Conclusion

The complexity of the ER is reflected by the intricacy of ER‐phagy processes. This field is relatively new and the mechanisms and roles of ER‐phagy have only recently begun to be unraveled. The number of ER‐phagy receptors is destined to continue to grow. New receptors will be identified by investigating ER proteins harboring functional LIR domains or ubiquitin‐interacting motifs, which are sequences that are able to bind mATG8s and recruit the initiation membranes 45. Uncovering the distinct molecular pathways that regulate wholesale ER degradation, versus the degradation of potions of the ER and/or ER protein aggregates selection is of great importance. Identifying and characterizing any auxiliary players, which may assist ER‐phagy receptors in ER turnover including chaperones and membrane shaping molecules, will be equally important. It is likely that ER‐phagy pathways are diverse and vary based on cell and tissue type. Finally, these pathways are implicated in the etiology of ER‐phagy–related diseases (i.e. sensory neuropathies or infectious diseases 6, 7, 10. Therefore, the study of the molecular mechanisms of ER‐phagy is likely holds the key to identify therapeutically relevant molecules that could act as potential druggable targets for the treatment of ER‐related disease.

## Conflict of interest

The authors declare no conflict of interest.

## Author contributions

AS and PG jointly wrote the manuscript.

## References

[1] Friedman JR & Voeltz GK (2011) The ER in 3D: a multifunctional dynamic membrane network. Trends Cell Biol 21, 709–17. 21900009 10.1016/j.tcb.2011.07.004 PMC3221873

[2] Nixon‐Abell J , Obara CJ , Weigel AV , Li D , Legant WR , Xu CS , Pasolli HA , Harvey K , Hess HF , Betzig E *et al* (2016) Increased spatiotemporal resolution reveals highly dynamic dense tubular matrices in the peripheral ER. Science 354, aaf3928(1–12). 10.1126/science.aaf3928 PMC6528812 27789813

[3] Voeltz GK , Prinz WA , Shibata Y , Rist JM & Rapoport TA (2006) A class of membrane proteins shaping the tubular endoplasmic reticulum. Cell 124, 573–586. 16469703 10.1016/j.cell.2005.11.047

[4] Phillips MJ & Voeltz GK (2016) Structure and function of ER membrane contact sites with other organelles. Nat Rev Mol Cell Biol 17, 69–82. 26627931 10.1038/nrm.2015.8 PMC5117888

[5] English AR , Zurek N & Voeltz GK (2009) Peripheral ER structure and function. Curr Opin Cell Biol 21, 596–602. 19447593 10.1016/j.ceb.2009.04.004 PMC2753178

[6] Wilkinson S (2019) Emerging principles of selective ER autophagy. J Mol Biol, pii: S0022-2836(19)30279-7. 10.1016/j.jmb.2019.05.012 PMC6971691 31100386

[7] Wilkinson S (2019) ER‐phagy: shaping up and destressing the endoplasmic reticulum. FEBS J. 10.1111/febs.14932 PMC6772018 31116513

[8] Smith M & Wilkinson S (2017) ER homeostasis and autophagy. Essays Biochem 61, 625–635. 29233873 10.1042/EBC20170092 PMC5869861

[9] Loi M , Fregno I , Guerra C & Molinari M (2018) Eat it right: ER‐phagy and recovER‐phagy. Biochem Soc Trans 46, 699–706. 29802216 10.1042/BST20170354 PMC6008593

[10] Grumati P , Dikic I & Stolz A (2018) ER‐phagy at a glance. J Cell Sci 131, pii: jcs217364. 10.1242/jcs.217364 30177506

[11] Fregno I & Molinari M (2018) Endoplasmic reticulum turnover: ER‐phagy and other flavors in selective and non‐selective ER clearance. F1000Res 7, 454. 29744037 10.12688/f1000research.13968.1 PMC5904726

[12] Fregno I , Fasana E , Bergmann TJ , Raimondi A , Loi M , Soldà T , Galli C , D'Antuono R , Morone D , Danieli A * et al* (2018) ER‐to‐lysosome‐associated degradation of proteasome‐resistant ATZ polymers occurs via receptor‐mediated vesicular transport. EMBO J 37, pii: e99259. 10.15252/embj.201899259 PMC6120659 30076131

[13] Dikic I (2018) Open questions: why should we care about ER‐phagy and ER remodelling? BMC Biol 16, 131. 30382915 10.1186/s12915-018-0603-7 PMC6211458

[14] Rogov V , Dötsch V , Johansen T & Kirkin V (2014) Interactions between autophagy receptors and ubiquitin‐like proteins form the molecular basis for selective autophagy. Mol Cell 53, 167–78. 24462201 10.1016/j.molcel.2013.12.014

[15] Locke M & Collins JV (1965) The structure and formation of protein granules in the fat body of an insect. J Cell Biol 26, 857–84. 19866685 10.1083/jcb.26.3.857 PMC2106788

[16] Bolender RP & Weibel ER (1973) A morphometric study of the removal of phenobarbital‐induced membranes from hepatocytes after cessation of threatment. J Cell Biol 56, 746–61. 4569312 10.1083/jcb.56.3.746 PMC2108940

[17] Tooze J , Hollinshead M , Ludwig T , Howell K , Hoflack B & Kern H (1990) In exocrine pancreas, the basolateral endocytic pathway converges with the autophagic pathway immediately after the early endosome. J Cell Biol 111, 329–345. 2166050 10.1083/jcb.111.2.329 PMC2116176

[18] Antonucci L , Fagman JB , Kim JY , Todoric J , Gukovsky I , Mackey M , Ellisman MH & Karin M (2015) Basal autophagy maintains pancreatic acinar cell homeostasis and protein synthesis and prevents ER stress. Proc Natl Acad Sci USA 112, E6166–E61744. 26512112 10.1073/pnas.1519384112 PMC4653219

[19] Pengo N , Scolari M , Oliva L , Milan E , Mainoldi F , Raimondi A , Fagioli C , Merlini A , Mariani E , Pasqualetto E *et al* (2013) Plasma cells require autophagy for sustainable immunoglobulin production. Nat Immunol 14, 298–305. 23354484 10.1038/ni.2524

[20] Jia W , Pua HH , Li QJ & He YW (2011) Autophagy regulates endoplasmic reticulum homeostasis and calcium mobilization in T lymphocytes. J Immunol 186, 1564–1574. 21191072 10.4049/jimmunol.1001822 PMC3285458

[21] Hamasaki M , Noda T , Baba M & Ohsumi Y (2005) Starvation triggers the delivery of the endoplasmic reticulum to the vacuole via autophagy in yeast. Traffic 6, 56–65. 15569245 10.1111/j.1600-0854.2004.00245.x

[22] Bernales S , McDonald KL & Walter P (2006) Autophagy counterbalances endoplasmic reticulum expansion during the unfolded protein response. PLoS Biol 4, e423. 17132049 10.1371/journal.pbio.0040423 PMC1661684

[23] Bernales S , Schuck S & Walter P (2007) ER‐phagy: selective autophagy of the endoplasmic reticulum. Autophagy 3, 285–287. 17351330 10.4161/auto.3930

[24] Schuck S , Prinz WA , Thorn KS , Voss C & Walter P (2009) Membrane expansion alleviates endoplasmic reticulum stress independently of the unfolded protein response. J Cell Biol 187, 525–536. 19948500 10.1083/jcb.200907074 PMC2779237

[25] Khaminets A , Heinrich T , Mari M , Grumati P , Huebner AK , Akutsu M , Liebmann L , Stolz A , Nietzsche S , Koch N *et al* (2015) Regulation of endoplasmic reticulum turnover by selective autophagy. Nature 522, 354–358. 26040720 10.1038/nature14498

[26] Mochida K , Oikawa Y , Kimura Y , Kirisako H , Hirano H , Ohsumi Y & Nakatogawa H (2015) Receptor‐mediated selective autophagy degrades the endoplasmic reticulum and the nucleus. Nature 522, 359–362. 26040717 10.1038/nature14506

[27] Chino H , Hatta T , Natsume T & Mizushima N (2019) Intrinsically disordered protein TEX264 Mediates ER‐phagy. Mol Cell 74, 909–921.e6. 31006538 10.1016/j.molcel.2019.03.033

[28] Chen Q , Xiao Y , Chai P , Zheng P , Teng J & Chen J (2019) ATL3 is a tubular ER‐phagy receptor for GABARAP‐mediated selective autophagy. Curr Biol 29, 846–855.e6. 30773365 10.1016/j.cub.2019.01.041

[29] An H , Ordureau A , Paulo JA , Shoemaker CJ , Denic V & Harper JW (2019) TEX264 is an endoplasmic reticulum‐resident ATG8‐interacting protein critical for ER remodeling during nutrient stress. Mol Cell 74, 891–908.e10. 31006537 10.1016/j.molcel.2019.03.034 PMC6747008

[30] Smith MD , Harley ME , Kemp AJ , Wills J , Lee M , Arends M , von Kriegsheim A , Behrends C & Wilkinson S (2018) CCPG1 is a non‐canonical autophagy cargo receptor essential for ER‐phagy and pancreatic ER proteostasis. Dev Cell 44, 217–232.e11. 29290589 10.1016/j.devcel.2017.11.024 PMC5791736

[31] Grumati P , Morozzi G , Hölper S , Mari M , Harwardt MI , Yan R , Müller S , Reggiori F , Heilemann M & Dikic I (2017) Full length RTN3 regulates turnover of tubular endoplasmic reticulum via selective autophagy. Elife 6, pii: e25555. 10.7554/eLife.25555 PMC5517149 28617241

[32] Fumagalli F , Noack J , Bergmann TJ , Cebollero E , Pisoni GB , Fasana E , Fregno I , Galli C , Loi M , Soldà T *et al* (2016) Translocon component Sec62 acts in endoplasmic reticulum turnover during stress recovery. Nat Cell Biol 18, 1173–1184. 27749824 10.1038/ncb3423

[33] Stolz A , Ernst A & Dikic I (2014) Cargo recognition and trafficking in selective autophagy. Nat Cell Biol 16, 495–501. 24875736 10.1038/ncb2979

[34] Deshaies RJ , Sanders SL , Feldheim DA & Schekman R (1991) Assembly of yeast Sec proteins involved in translocation into the endoplasmic reticulum into a membrane‐bound multisubunit complex. Nature 349, 806–808. 2000150 10.1038/349806a0

[35] Hu J , Shibata Y , Zhu PP , Voss C , Rismanchi N , Prinz WA , Rapoport TA & Blackstone C (2009) A class of dynamin‐like GTPases involved in the generation of the tubular ER network. Cell 138, 549–561. 19665976 10.1016/j.cell.2009.05.025 PMC2746359

[36] Bhaskara RM , Grumati P , Garcia‐Pardo J , Kalayil S , Covarrubias‐Pinto A , Chen W , Kudryashev M , Dikic I & Hummer G (2019) Curvature induction and membrane remodeling by FAM134B reticulon homology domain assist selective ER‐phagy. Nat Commun 10, 2370. 31147549 10.1038/s41467-019-10345-3 PMC6542808

[37] Forrester A , DeLeonibus C , Grumati P , Fasana E , Piemontese M , Staiano L , Fregno I , Raimondi A , Marazza A , Bruno G *et al* (2019) A selective ER‐phagy exerts procollagen quality control via a Calnexin‐FAM134B complex. EMBO J 38, pii: e99847. 10.15252/embj.201899847 PMC6331724 30559329

[38] Cunningham CN , Williams JM , Knupp J , Arunagiri A , Arvan P & Tsai B (2019) Cells deploy a two‐pronged strategy to rectify misfolded proinsulin aggregates. Mol Cell, pii: S1097-2765(19)30364-8. 10.1016/j.molcel.2019.05.011 PMC6688957 31176671

[39] Meszaros B , Tompa P , Simon I & Dosztányi Z (2007) Molecular principles of the interactions of disordered proteins. J Mol Biol 372, 549–561. 17681540 10.1016/j.jmb.2007.07.004

[40] Liang JR , Lingeman E , Ahmed S & Corn JE (2018) Atlastins remodel the endoplasmic reticulum for selective autophagy. J Cell Biol 217, 3354–3367. 30143524 10.1083/jcb.201804185 PMC6168278

[41] Chen S , Cui Y , Parashar S , Novick PJ & Ferro‐Novick S (2018) ER‐phagy requires Lnp1, a protein that stabilizes rearrangements of the ER network. Proc Natl Acad Sci USA 115, E6237–E6244. 29915089 10.1073/pnas.1805032115 PMC6142256

[42] Cui Y , Parashar S , Zahoor M , Needham PG , Mari M , Zhu M , Chen S , Ho HC , Reggiori F , Farhan H *et al* (2019) A COPII subunit acts with an autophagy receptor to target endoplasmic reticulum for degradation. Science 365, 53–60. 31273116 10.1126/science.aau9263 PMC7062386

[43] Duan G & Walther D (2015) The roles of post‐translational modifications in the context of protein interaction networks. PLoS Comput Biol 11, e1004049. 25692714 10.1371/journal.pcbi.1004049 PMC4333291

[44] van Klompenburg W , Nilsson I , vonHeijne G & de Kruijff B (1997) Anionic phospholipids are determinants of membrane protein topology. EMBO J 16, 4261–4266. 9250669 10.1093/emboj/16.14.4261 PMC1170051

[45] Marshall RS , Hua Z , Mali S , McLoughlin F & Vierstra RD (2019) ATG8‐binding UIM proteins define a new class of autophagy adaptors and receptors. Cell 177, 766–781.e24. 30955882 10.1016/j.cell.2019.02.009 PMC6810650

